# Primary Thromboprophylaxis in Ambulatory Pancreatic Cancer Patients Receiving Chemotherapy: A Systematic Review and Meta-Analysis of Randomized Controlled Trials

**DOI:** 10.3390/cancers12082028

**Published:** 2020-07-24

**Authors:** Corinne Frere, Benjamin Crichi, Barbara Bournet, Cindy Canivet, Nassim Ait Abdallah, Louis Buscail, Dominique Farge

**Affiliations:** 1 Institute of Cardiometabolism and Nutrition, INSERM UMRS_1166, GRC 27 GRECO, Sorbonne Université, F-75013 Paris, France; corinne.frere@aphp.fr; 2 Department of Hematology, Pitié-Salpêtrière Hospital, Assistance Publique Hôpitaux de Paris, F-75013 Paris, France; 3 Internal Medicine, Autoimmune and Vascular Disease Unit, Saint-Louis Hospital, Assistance Publique Hôpitaux de Paris, F-75010 Paris, France; benjamin.crichi@aphp.fr (B.C.); nassim.aitabdallah@aphp.fr (N.A.A.); 4 Department of Gastroenterology and Pancreatology, University of Toulouse, F-31000 Toulouse, France; bournet.b@chu-toulouse.fr (B.B.); canivet.c@chu-toulouse.fr (C.C.); buscail.l@chu-toulouse.fr (L.B.); 5 Department of Gastroenterology and Pancreatology, CHU de Toulouse, F-31300 Toulouse, France; 6 Institut Universitaire d’Hématologie, Université de Paris, EA 3518, F-75010 Paris, France; 7 Department of Medicine, McGill University, Montreal, QC H4A 3J1, Canada

**Keywords:** pancreatic cancer, venous thromboembolism, thromboprophylaxis, anticoagulants, chemotherapy, major bleeding

## Abstract

Patients with pancreatic cancer (PC) carry the highest risk of venous thromboembolism (VTE) amongst all cancer patients. Appropriate use of primary thromboprophylaxis might significantly and safely reduce its burden. We performed a systematic review of published studies and meeting abstracts using MEDLINE and EMBASE through July 2020 to evaluate the efficacy and safety of primary thromboprophylaxis in ambulatory PC patients receiving chemotherapy. The Mantel–Haenszel random effect model was used to estimate the pooled event-based risk ratio (RR) and the pooled absolute risk difference (RD) with a 95% confidence interval (CI). Five randomized controlled studies with 1003 PC patients were included in this meta-analysis. Compared to placebo, thromboprophylaxis significantly decreased the risk of VTE (pooled RR 0.31, 95% CI 0.19–0.51, *p* < 0.00001, I^2^ = 8%; absolute RD −0.08, 95% CI −0.12–−0.05, *p* < 0.00001, I^2^ = 0%), with an estimated number needed to treat of 11.9 patients to prevent one VTE event. Similar reductions of VTE were observed in studies with parenteral (RR 0.30, 95% CI 0.17–0.53) versus oral anticoagulants (RR 0.37, 95% CI 0.14–0.99) and in studies using prophylactic doses of anticoagulants (RR 0.34, 95% CI 0.17–0.70) versus supra-prophylactic doses of anticoagulants (RR 0.27, 95% CI 0.08–0.90). The pooled RR for major bleeding was 1.08 (95% CI 0.47–2.52, *p* = 0.85, I^2^ = 0%) and the absolute RD was 0.00 (95% CI −0.02–0.03, *p* = 0.85, I^2^ = 0%). Evidence supports a net clinical benefit of thromboprophylaxis in ambulatory PC patients receiving chemotherapy. Adequately powered randomized phase III studies assessing the most effective anticoagulant and the optimal dose, schedule and duration of thromboprophylaxis to be used are warranted.

## 1. Introduction

Venous thromboembolism (VTE) is a common complication and the second most common preventable cause of death in cancer patients. Individuals with pancreatic cancer (PC) have been shown to carry the highest risk of VTE of any cancer type [1,2], with VTE rates ranging from 5% to 41% in retrospective cohorts [3,4,5,6,7,8,9,10,11,12,13,14,15,16,17,18,19,20], and up to 67% in postmortem series [21]. In a recent large prospective cohort study of 731 PC patients, VTE occurred in 21% of patients and was associated with significant decreases in both progression-free survival (PFS) and overall survival (OS) [22].

Most clinical practice guidelines (CPG) now recommend thromboprophylaxis for ambulatory cancer patients at high risk of VTE, in the absence of contra-indication [23,24]. In ambulatory advanced PC patients receiving chemotherapy, the benefit of low molecular weight heparin (LMWH) for preventing VTE has been initially established in two dedicated randomized controlled trials (RCT), demonstrating that LMWH reduces the rate of VTE by 66–85%, without increasing the risk of major bleeding [25,26]. Based on these findings, the International Initiative on Cancer and Thrombosis (ITAC) CPG have recommended the use of LMWH in these patients since 2013, provided they have a low risk of bleeding (Grade 1B) [23,27,28]. However, both the burden of VTE and the benefit of primary thromboprophylaxis in PC patients continue to be under-recognized worldwide, and implementation of this CPG remains insufficient.

Recently, the CASSINI trial assessed the efficacy and safety of prophylactic doses of rivaroxaban for preventing VTE in ambulatory cancer patients receiving systemic anticancer therapy who were at intermediate-to-high-risk of VTE (Khorana score ≥ 2 prior to starting chemotherapy) [29]. A subgroup analysis of PC patients included in the CASSINI phase III trial was subsequently released [29,30]. This subgroup analysis increases the size and quality of the dataset available for a meta-analysis assessing the efficacy and safety of anticoagulants for primary thromboprophylaxis in PC patients. Careful comparison of these results with those from previous studies on this topic is warranted since the use of direct oral anticoagulants (DOAC) might improve the net clinical benefit of thromboprophylaxis in PC patients.

Herein, we report a systematic literature review and meta-analysis of all RCTs assessing the benefit of anticoagulants versus placebo or non-placebo control for the prevention of VTE in ambulatory PC patients receiving systemic chemotherapy. We performed sensitivity analyses regarding parenteral versus oral anticoagulants and prophylactic versus supra-prophylactic doses, in order to determine the optimal anticoagulant agent and dosing to be used for primary thromboprophylaxis.

## 2. Results

### 2.1. Search Results and Study Characteristics

A total of 2156 records were identified through database searching. After title and abstract screening, 6 studies were assessed for eligibility [25,26,29,30,31,32,33]. One study did not report data separately from the subgroup of PC patients [33]. Finally, 5 studies meeting the inclusion criteria were included in the meta-analysis (Figure S1) [25,26,29,30,31,32]. The agreement between reviewers for study selection was 100% (kappa statistic 1.0), and no further resolution by a third reviewer was required. Data were available from 2 dedicated RCTs [25,26] and from 3 other RCTs which reported results from a subgroup of PC patients [29,31,32]. In total, data from 1003 PC participants were analyzed.

The characteristics of each study are depicted in Table 1. The study design, dosing and duration of primary thromboprophylaxis widely differed between trials. Two dedicated open-label phase IIb RCTs (FRAGEM [25] and CONKO-004 [26]) evaluated the efficacy and safety of primary thromboprophylaxis with either dalteparin at therapeutic doses (dalteparin 200 International Units [IU]/kg once daily for 4 weeks, followed 150 IU/kg) [25] or enoxaparin at supra-prophylactic doses (enoxaparin 1 mg/kg once daily) [26] in ambulatory patients with advanced PC receiving chemotherapy, with no treatment as a comparator. Three double-blinded placebo-controlled phase III RCTs evaluated the efficacy and safety of primary thromboprophylaxis with prophylactic doses of nadroparin (3800 IU once daily, PROTECHT trial [31]) or semuloparin (20 mg once daily, SAVE-ONCO trial [32]) or rivaroxaban (10 mg once daily, CASSINI trial [29,30]) in ambulatory patients with various cancer types receiving chemotherapy and reported data from a subgroup of PC patients. Duration of thromboprophylaxis ranged from 3 to 6 months (Table 1). Systematic VTE screening was performed in only 1 study [29]. Three studies were classified as good quality according to the JADAD scale (Table S1) and the overall risk of bias was considered low according to the Cochrane risk assessment tool (Figures S2 and S3).

### 2.2. Efficacy of Primary Thromboprophylaxis with Anticoagulants

**Figure 1:**
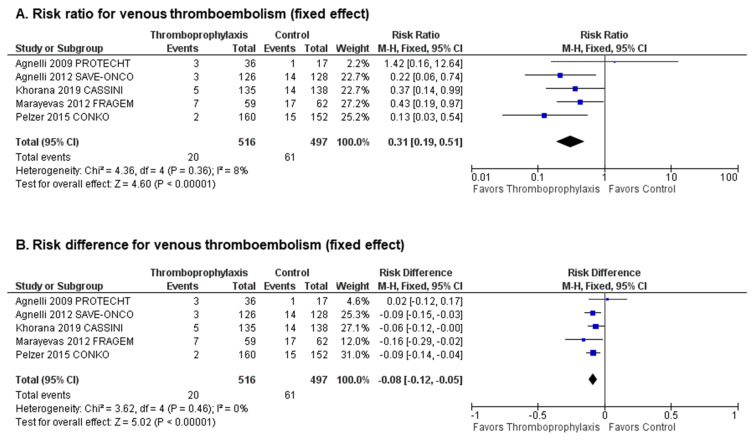
Efficacy analysis: Forest plots of (**A**) risk ratios (RR) and (**B**) risk differences (RD) for venous thromboembolism (VTE).

Together, the 5 studies reported a total of 81 VTE events (20 in patients receiving primary thromboprophylaxis and 61 in patients receiving placebo or no treatment) [25,26,29,30,31,32]. The overall incidence rate of VTE was significantly lower in PC patients receiving primary thromboprophylaxis (3.87%) relative to placebo or no treatment (12.27%). Compared to control, primary thromboprophylaxis significantly decreased the risk of VTE (pooled Risk Ratio (RR) 0.31, 95% CI (confidence interval) 0.19–0.51, *p* < 0.00001, I^2^ = 8%, Figure 1B; absolute Risk Difference (RD) −0.08, 95% CI −0.12–−0.05, *p* < 0.00001, I^2^ = 0%, Figure 1B), with an estimated number needed to treat (NNT) of 11.9 patients to prevent one VTE event. Visual inspection of funnel plots showed no evidence of publication bias (Figure S4).

Primary prophylaxis with parenteral anticoagulants (4 studies [25,26,31,32], 740 patients; RR 0.30; 95% CI 0.17–0.53, I^2^ = 31%) or oral anticoagulants (1 study [30], 273 patients; RR 0.37; 95% CI 0.14–0.99) reduced the risk of VTE with the same magnitude (Table 2). VTE risk reduction was similar in the 3 placebo-controlled studies with prophylactic doses of anticoagulants [29,31,32] (580 patients; RR 0.34; 95% CI 0.17–0.70, I^2^ = 7%) and in the 2 studies with supra-prophylactic or therapeutic doses of anticoagulants as compared to no treatment [25,26] (433 patients; RR 0.27; 95% CI 0.08–0.90, I^2^ = 55%, Table 2).

### 2.3. Safety of Primary Thromboprophylaxis with Anticoagulants

**Figure 2:**
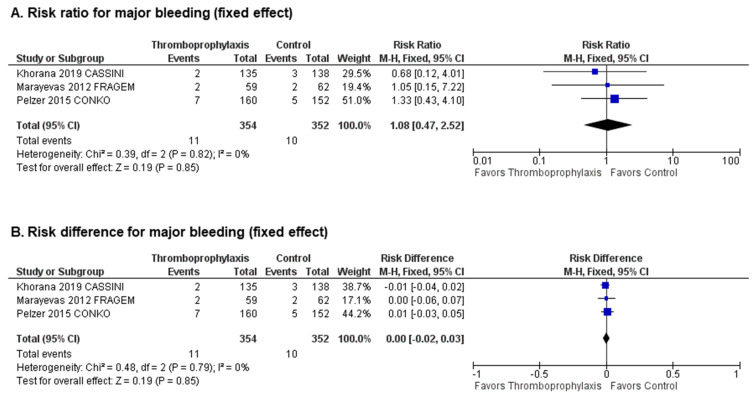
Safety analysis: Forest plots of (**A**) risk ratios and (**B**) risk differences for major bleeding.

Three studies [25,26,30] reported a total of 21 major bleeding events (11 in patients receiving primary thromboprophylaxis and 10 in patients receiving placebo or no treatment). The overall incidence of major bleeding was similar between patients receiving primary thromboprophylaxis (3.10%) and those receiving placebo or no treatment (2.84%). A pooled analysis demonstrated no statistically significant increase in the risk of major bleeding with the use of parenteral or oral primary thromboprophylaxis (pooled RR 1.08; 95% CI 0.47–2.52, *p* = 0.85, I^2^ = 0%, Figure 2A; absolute RD 0.00; 95% CI −0.02–0.03, *p* = 0.85, I^2^ = 0%, Figure 2B) with an estimated number needed to harm (NNH) of 384.6 patients to avoid one major bleeding. Visual inspection of funnel plots showed no evidence of publication bias (Figure S5).

Similarly, in sensitivity analyses, there was no significant increase in the risk of major bleeding with primary thromboprophylaxis in studies using parenteral anticoagulants (2 studies [25,26], 433 patients; RR 1.25; 95% CI 0.47–3.31, I^2^ = 0%) or oral anticoagulants (1 study [30], 273 patients; RR 0.68; 95% CI 0.12–4.01), and in studies using prophylactic doses of anticoagulants (1 study [30], 273 patients; RR 0.68; 95% CI 0.12–4.01) or supra-prophylactic or therapeutic doses of anticoagulants (2 studies [25,26], 433 patients; RR 1.25; 95% CI 0.47–3.31, I^2^ = 0%) (Table 2).

### 2.4. Net Clinical Benefit of Primary Thromboprophylaxis with Anticoagulants

**Figure 3:**
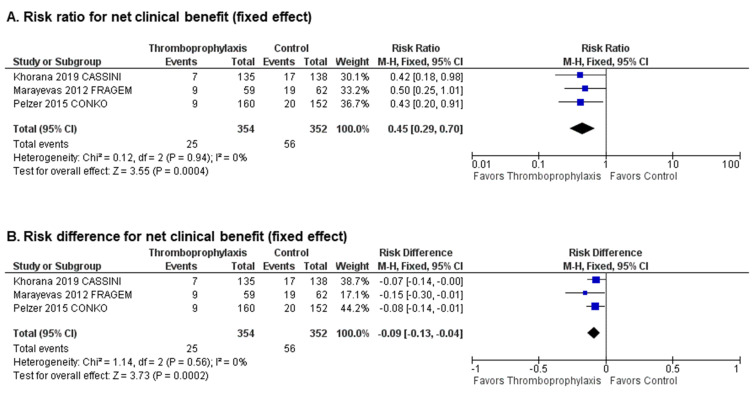
Net clinical benefit: Forest plots of (**A**) risk ratios and (**B**) risk differences for net clinical benefit.

Pooled analysis demonstrated that primary thromboprophylaxis significantly improves the net clinical benefit (pooled RR 0.45; 95% CI 0.29–0.70, *p* = 0.0004, I^2^ = 0%, Figure 3A; absolute RD −0.09; 95% CI −0.13–−0.04, *p* = 0.0002, I^2^ = 0%, Figure 3B).

## 3. Discussion

The present meta-analysis pooled data from 1003 PC patients enrolled in 5 RCTs that compared anticoagulants (parenteral or oral) with placebo or no placebo control for primary VTE prevention in cancer patients receiving chemotherapy. Overall, primary thromboprophylaxis with anticoagulants was found to be associated with a 69% relative risk reduction in the rates of VTE without heterogeneity between studies, resulting in a NNT of 11.9 to prevent one VTE event. Primary thromboprophylaxis exhibited a significant net clinical benefit by drastically decreasing the risk of VTE without increasing the risk for major bleeding.

A previous Cochrane meta-analysis which assessed the benefit of primary LMWH thromboprophylaxis in unselected ambulatory cancer patients receiving chemotherapy reported that LMWH significantly reduced the rate of VTE (RR 0.54, 95% CI 0.38–0.75) with a non-statistically significant increase in the risk of major bleeding events (RR 1.44, 95% CI 0.98–2.11) [34]. In this meta-analysis, the NNT appeared too high to support the use of thromboprophylaxis in all ambulatory cancer patients, due to an overall low rate of VTE events in the study population [34]. Therefore, it was suggested to rather use targeted thromboprophylaxis in high-risk patients. A risk-stratified approach based on the Khorana score [35] has been proposed to select patients at high risk of VTE. However, it is questionable whether its use is relevant for PC patients given that the Khorana score assigns +2 points for PC, thereby classifying all PC patients at intermediate risk of VTE, at least. It is therefore not surprising that the Khorana score has low predictive power in determining differences in VTE risk within the PC patient population, both in a small retrospective study of PC patients undergoing chemotherapy [36], and more recently in the large prospective Base Clinico-Biologique de l’Adénocarcinome Pancréatique [BACAP]-VTE study [5], the Khorana score failed to accurately predict VTE risk across PC patients. Nevertheless, the overall high incidence of VTE in PC patients has prompted recent CPGs to recommend that primary thromboprophylaxis should be considered in all PC patients undergoing chemotherapy who are at low risk of bleeding [23,24].

Indeed, compared to other cancer patients, PC patients carry a higher risk of VTE [1,2], with reported incidence rates of VTE ranging from 5% to 41% in retrospective cohorts, depending on the study population [3,4,5,6,7,8,9,10,11,12,13,14,15,16,17,18,19,20]. In the largest prospective multicenter cohort of patients with newly diagnosed PC to date [22], VTE occurred in more than 20% of patients, with a median time from PC diagnosis to VTE of 4.49 months, highlighting the need for an adequate thromboprophylaxis scheme in these patients. Moreover, PC patients who developed VTE had significantly shorter PFS and OS compared to those without VTE, even after adjustment for age and cancer stage [22].

Our study further confirms the results of a previous meta-analysis [37] demonstrating a clear benefit of LMWH primary thromboprophylaxis in PC patients with the addition of a subgroup of 273 PC patients enrolled in the CASSINI trial, which recently demonstrated the benefit of rivaroxaban in preventing VTE in intermediate-to-high-risk cancer patients (Khorana score ≥ 2) [29,30].

To our knowledge, this is the first systematic review and meta-analysis assessing the efficacy and safety of thromboprophylaxis in the PC patient population that has included data from a subgroup of PC patients enrolled in a DOAC placebo-controlled trial for primary VTE prevention. The double-blind placebo-controlled CASSINI trial randomized cancer patients initiating chemotherapy and classified as intermediate-to-high-risk of VTE (as defined by a Khorana score ≥ 2) to receive either primary prophylaxis with 10 mg rivaroxaban or placebo, once daily, for up to 6 months [29]. A pre-specified subgroup analysis of PC patients reported that the primary composite endpoint (deep vein thrombosis [DVT], asymptomatic proximal DVT, pulmonary embolism [PE] and VTE-related death within the first 180 days after randomization) occurred in 5 out of 135 PC patients in the rivaroxaban arm compared to 14 out of 138 (10.1%) patients in the placebo arm, without difference in major bleeding between the two arms [30]. The cumulative incidence rates of VTE in the placebo and thrombopropylaxis groups at 6 months were consistent with those observed in the PROSPECT-CONKO 004 trial [26] and in the subgroup analysis of PC patients included in the SAVE-ONCO trial [32]. In sensitivity analyses, similar reductions in VTE rates were observed when using parenteral (LMWH) or oral anticoagulants (DOAC), suggesting that DOAC might be as efficient as LMWH for VTE prevention in this specific population. However, while parenteral anticoagulants were used in a total of 740 patients included in 4 studies, only a single subgroup of 273 PC patients from a non-PC DOAC placebo-controlled trial was included in our meta-analysis, which may have introduced a bias in the sensitivity analysis. Therefore, these results should be interpreted with caution and more data from dedicated trials are needed to assess the benefit of DOAC in this specific setting and confirm this finding.

Primary thromboprophylaxis with LMWH in advanced or metastatic PC patients receiving systemic anticancer therapy who are at low risk of bleeding (Grade 1B) has been recommended by the ITAC CPGs since 2013 [23,27,28]. However, fear of bleeding in otherwise frail patients, the inconvenience of prolonged parenteral therapy and the inherent costs for such therapy remain major concerns for both patients and physicians. DOAC, if shown to have similar efficacy and safety profiles, can present an alternative to LMWH that would address the potential treatment burden associated with administering daily injections of a parenteral anticoagulant for extended periods, and improve the feasibility of thromboprophylaxis in PC patients. Nonetheless, the use of DOAC in these patients has potential limitations that should be taken into consideration to guide treatment choice. First, data on their efficacy and safety in patients with extreme body weights (i.e., cachexia or obesity) are lacking and the use of DOAC in these patients may result in over- or under-coagulation. Second, DOAC should be used with caution in the elderly who have been shown to be at high risk of bleeding. Third, careful consideration of competing risks, which include presence of comorbidities (e.g., renal or hepatic impairment), drug–drug interactions that may affect DOAC pharmacokinetics and chemotherapy common side effects (vomiting and diarrhea), that can limit DOACs absorption, is warranted. Therefore, an individualized approach is necessary, and for each single PC patient, full consideration of the appropriate balance of benefits and harms is important.

It has been suggested that PC patients receiving chemotherapy may require higher doses of anticoagulant for VTE prevention since dalteparin was administered at therapeutic doses in the FRAGEM trial [25]. On the other hand, enoxaparin was administered at supra-prophylactic doses in the PROSPECT-CONKO 004 trial [26]. In the present meta-analysis, similar VTE risk reductions were observed using either therapeutic, prophylactic or supra-prophylactic doses of anticoagulant, suggesting that the use of higher doses of anticoagulant does not improve the net clinical benefit of primary thromboprophylaxis. Once again, these results need to be interpreted with caution since sensitivity analyses of prophylactic doses were performed in subgroups of PC patients from non-PC anticoagulant placebo-controlled trials [29,30,31,32].

The optimal duration of primary thromboprophylaxis in PC patients receiving chemotherapy remains an unanswered question. Thromboprophylaxis duration ranged from 3 to 6 months in RCTs included in this meta-analysis and we did not observe any difference in VTE risk reduction between studies using a 3- or 6-month duration of thromboprophylaxis. Whether longer periods of prophylaxis can be of benefit with a similar safety profile remains unknown.

The prevalence of PC is projected to increase by approximately 40% over the next decade in North America and Europe [38]. Despite recent advancements in the management of PC patients, the prognosis remains poor, with few patients surviving to 10 years [39]. Therefore, there is an urgent need for optimizing and integrating supportive care, especially VTE prevention, which will improve patient quality of life and, potentially, OS [36,40].

Despite a significant association between VTE and mortality in PC patients, the FRAGEM [25] and PROSPECT-CONKO 004 [26] failed to demonstrate a benefit of LMWH on overall survival, which might be related to the short life expectancy of patients included in these studies [41]. In the FRAGEM trial, 3 out of 4 deaths observed in the placebo arm in the first 3 months of treatment were secondary to VTE, compared to only one death in the dalteparin arm, which was due to sepsis [25]. A non-randomized trial reported that the use of nadroparin improved survival in 69 consecutive patients with advanced PC treated with Gemcitabine plus cisplatin every 21 days until disease progression. The overall response total response rate was 58.8% with nadroparin compared to 12.1% without nadroparin (*p* = 0.0001). Patients receiving nadroparin had longer PFS (7.3 versus 4.0 months, *p* = 0.0001) and OS (13.0 versus 5.5 months, *p* = 0.0001) compared to those not receiving nadroparin [42]. Future studies to determine the role of LMWH or DOAC thromboprophylaxis in improving overall cancer-related treatment outcomes are required.

Our study has several limitations inherent to meta-analyses. First, selected studies were heterogeneous in terms of study design, study population, primary thromboprophylaxis modalities (e.g., anticoagulant agent, dosing, schedule, duration of thromboprophylaxis), VTE outcome definitions and length of follow-up. Second, data were derived from subgroup analyses of PC patients in 3 out of the 5 studies included in our meta-analysis. Finally, the analysis was not based on individual data.

In the absence of head-to-head comparison between LMWH and DOACs, it is not yet possible to conclude on the superiority of one agent over the other. Only an adequately powered randomized, double-blind, DOAC-LMWH-controlled, non-inferiority multicenter trial with a 6-month follow-up duration would allow to draw definitive conclusions.

## 4. Materials and Methods

This systematic review and meta-analysis were performed in accordance with the Preferred Reporting Items for Systematic Reviews and Meta-Analyses (PRISMA) guidelines (http://www.prisma-statement.org/) [43].

### 4.1. Study Objectives

The primary objectives were to assess the efficacy and the safety of pharmacological primary prophylaxis using either parenteral (LMWH) or oral (vitamine K antagonists [VKA] and DOAC) anticoagulant drugs for VTE prophylaxis in ambulatory PC patients receiving chemotherapy. The secondary objective was to assess the net clinical benefit of pharmacological primary prophylaxis in these patients.

### 4.2. Study Outcomes

The primary efficacy outcome was the overall incidence of VTE including symptomatic pulmonary embolism (PE), symptomatic deep vein thrombosis (DVT), incidental PE, asymptomatic ultrasound-detected DVT and VTE-related death adjudicated according to the criteria of the individual RCT during the entire follow-up. The primary safety outcome was the overall incidence of major bleeding defined according to the criteria of the individual RCT. The secondary outcome was the net clinical benefit defined as a composite of the rate of VTE and major bleeding, reflecting the overall effect of anticoagulants.

### 4.3. Literature Search

We performed a comprehensive literature search of published studies and meeting abstracts from all languages using MEDLINE (from 1946) and EMBASE (from 1947) through July 2020. We also hand-searched annual meeting abstracts of American Society of Hematology, American Society of Clinical Oncology and International Society on Thrombosis and Hemostasis. In addition, we searched for unpublished studies on clinicaltrials.gov. The search strategy included the terms “(((“Cancer”) AND ((((“Venous Thromboembolism”) OR “Thrombosis”) OR “Venous Thrombosis”) OR “Thromboembolism”))) AND (“prevention and control” OR “anticoagulant” OR “low molecular weight heparin (LMWH)” OR “warfarin” OR “vitamin K antagonist” (VKA) OR “Heparin” OR “dalteparin” OR “enoxaparin” OR “apixaban” OR “rivaroxaban” OR “edoxaban” or “dabigatran”) and further, was limited to RCTs.

### 4.4. Study Eligibility

Studies were eligible to be included in the meta-analysis if the initial screening met the inclusion criteria of (1) RCTs, (2) comparing anticoagulant (LMWH or VKA or DOAC) versus placebo or non-placebo control for primary prevention of VTE, (3) enrolling ambulatory PC patients receiving chemotherapy and (4) having a follow-up of at least three months. Two reviewers (C.F. and B.C.) independently screened all records identified in the literature search for study eligibility based on title and abstracts. In cases of duplicate publications, the most recent publication was considered. The agreement between the reviewers for study selection was assessed using the kappa statistic [44]. Any discrepancies were resolved by consensus after discussion between the two authors (C.F. and B.C.) and adjudicated by a third author (D.F.) when necessary. Only studies with extractable data on PC patients were included in the final analysis.

### 4.5. Data Extraction

Data were independently extracted using dedicated forms by both reviewers. The data collected included general data (authors, year of publication, study design), baseline patients’ characteristics (number, mean age, gender), study procedures (randomization process, treatment allocation, blinding process), anticoagulant prophylaxis (agent, regimen, duration), follow-up (duration, screening for VTE, lost to follow-up) and clinical outcomes (overall VTE, symptomatic VTE, VTE-related death and major bleeding).

### 4.6. Study Quality and Assessment of Risk of Bias

We used the JADAD score [45] and the Cochrane Risk of Bias Tool for clinical trials (sequence generation, allocation concealment, blinding, detection bias, attrition bias and reporting bias) to assess the quality and risk of bias in the included studies [46].

### 4.7. Statistical Analysis

Mantel–Haenszel random-effects and fixed-effects models (Der Simonian–Laird analysis [47]) were used to estimate the pooled event-based RR and the pooled absolute RD, with 95% CI in the presence or absence of significant heterogeneity, respectively. Heterogeneity among studies was assessed by the I^2^ statistic, with I^2^ > 50% representing a high degree of heterogeneity. Visualization of funnel plots was used to assess for publication bias. Sensitivity analyses were performed to compare (1) parenteral versus oral anticoagulants, and (2) prophylactic versus supra-prophylactic/therapeutic doses of anticoagulants. Statistical analysis was performed using the Cochrane’s Review Manager software (RevMan, version 5.3, Copenhagen, Denmark). The NTT was calculated as previously described [48], i.e., NNT = 1/absolute risk reduction. A *p*-value < 0.05 was considered as statistically significant.

## 5. Conclusions

In conclusion, the present study demonstrates that appropriate use of primary thromboprophylaxis, using either LMWH or DOAC, can significantly and safely reduce the burden of VTE in PC patients. Increased awareness among healthcare professionals and adherence to evidence-based CPGs will improve the care of these fragile patients. In the absence of head-to-head comparison between LMWH and DOAC, careful consideration of competing risks in each individual patient and discussion with the patient about the relative benefits and risks, drug cost, duration and tolerance of each anticoagulant are warranted.

## Figures and Tables

**Table 1:** Characteristics of the studies included in the metanalysis.

Study	Study Type	Number of PC Patients (Intervention /Control)	Patient Characteristics	Study Treatments	Duration of Thromboprophylaxis	Primary Endpoint	Efficacy Outcome		Major Bleeding	
							Intervention	Control	Intervention	Control
Agnelli 2009 PROTECHT [31]	Randomized, double blind, placebo-controlled multicenter phase III study	36/17	Ambulatory patients aged 18 years or older on chemotherapy with metastatic or locally advanced PC, ECOG Performance Status ≤ 2	Nadroparin (3800 IU o.d.) versus placebo	For the duration of chemotherapy up to a maximum of 4 months	Composite of symptomatic VTE or arterial thromboembolism	3/36	1/17	-	-
Agnelli 2012SAVE ONCO [32]	Randomized, double blind, placebo-controlled multicenter phase III study	126/128	Ambulatory patients aged 18 years or older with metastatic or locally advanced PC beginning to receive a course of chemotherapy, ECOG PS < 3	Semuloparin (20 mg o.d.) versus placebo	For the duration of chemotherapy then discontinued when chemotherapy was stopped, or regimen changed	Any symptomatic DVT in lower or upper limbs, any non-fatal PE, or death related to VTE (fatal PE or unexplained death)	3/126	14/128	-	-
Marayevas 2012FRAGEM [25]	Randomized, open label, controlled Phase IIb study	59/62	Patients aged 18 years or older with advanced or metastatic PC, Karnofsky performance status 60–100	Gemcitabine + Dalteparin (200 IU/kg o.d., for 4 weeks, followed by a step-down regimen to 150 IU/kg) versus Gemcitabine alone	For up to 12 weeks	All type DVT/PE, all arterial events and all visceral thromboembolism	7/59	17/62	2/59	2/62
Pelzer 2015 CONKO [26]	Prospective, open label, randomized, multicenter and group-sequential phase IIb study	160/152	Patients aged 18 years or older with advanced PC receiving ambulant first-line chemotherapy	Enoxaparin 1 mg/kg o.d. versus no enoxaparin	Until disease progression	First symptomatic VTE	2/160	15/152	7/160	5/152
Khorana 2019 CASSINI [29,30]	Double-blind, randomized, placebo-controlled, parallel-group, multicenter phase III study	135/138	Adult ambulatory patients with various cancers initiating a new systemic regimen and at increased risk for VTE (defined as Khorana score ≥ 2).	Rivaroxaban 10 mg o.d. versus placebo	180 (±3) days	Objectively confirmed symptomatic or asymptomatic lower-extremity proximal DVT, symptomatic upper extremity or distal lower-extremity DVT, symptomatic or incidental PE and VTE–related death	5/135	14/138	2/135	3/138

Abbreviations: DVT, deep vein thrombosis; ECOG, Eastern Cooperative Oncology Group; IU, international unit; o.d., once daily; PC, pancreatic cancer; PE, pulmonary embolism; VTE, venous thromboembolism.

**Table 2:** Results of sensitivity analyses.

**Sensitivity Analyses of Efficacy**	***n*** **Studies, *n* Patients**	**RR (95% CI)**	***p*** **(Test for Overall Effect)**	**I² (%)**
Parenteral anticoagulants [25,26,31,32]	4 studies, 740 patients	0.30 (0.17–0.53)	<0.0001	31
Oral anticoagulants [30]	1 study, 273 patients	0.37 (0.14–0.99)	0.05	NA
Prophylactic doses of anticoagulants [30,31,32]	3 studies, 580 patients	0.34 (0.17–0.70)	0.003	7
Supra-prophylactic or therapeutic doses of anticoagulants [25,26]	2 studies, 433 patients	0.27 (0.08–0.90)	0.03	55
**Sensitivity Analyses of Safety**	***n*** **Studies,** ***n*** **Patients**	**RR (95% CI)**	***p*** **(Test for Overall Effect)**	**I² (%)**
Parenteral anticoagulants [25,26]	2 studies, 433 patients	1.25 (0.47–3.31)	0.65	0
Oral anticoagulants [30]	1 study, 273 patients	0.68 (0.12–4.01)	0.67	NA
Prophylactic doses of anticoagulants [30]	1 study, 273 patients	0.68 (0.12–4.01)	0.67	NA
Supra-prophylactic or therapeutic doses of anticoagulants [25,26]	2 studies, 433 patients	1.25 (0.47–3.31)	0.65	0

Abbreviations: NA, not applicable; RR, risk ratio; 95% CI, 95% confidence interval.

## Supplementary Materials

The following are available online at https://www.mdpi.com/2072-6694/12/8/2028/s1, Table S1: Quality assessment of included studies according to the JADAD score, Figure S1: Search Flow Diagram, Figure S2: Quality assessment of included studies according to the Cochrane risk assessment tool, Figure S3: Risk of bias presented as percentages across all included studies according to the Cochrane risk assessment tool, Figure S4: Funnel plot for venous thromboembolism, Figure S5: Funnel plot for major bleeding.
