# Supplementary material for: Primary Thromboprophylaxis in Ambulatory Pancreatic Cancer Patients Receiving Chemotherapy: A Systematic Review and Meta-Analysis of Randomized Controlled Trials
Source: Cancers (Basel). 2020 Jul 24;12(8):2028. doi: 10.3390/cancers12082028 (PMC7464699; doi:10.3390/cancers12082028)
Supplement: Supplementary file 1 [file cancers-12-02028-s001.pdf]

Supplementary Materials: Primary Thromboprophylaxis in Ambulatory Pancreatic Cancer Patients receiving Chemotherapy: A systematic Review and Meta-analysis of Randomized Controlled Trials.

Corinne Frere, Benjamin Crichi, Barbara Bournet, Cindy Canivet, Nassim Ait Abdallah, Louis Buscail and Dominique Farge

Table S1. Quality assessment of included studies according to the Jadad score.

Author, Year, Study	Randomization	Appropriate randomization scheme	Double-blind	Appropriate double blinding	Description of dropouts and withdrawals	Sum score
**Agnellli 2009 PROTECHT**	1	1	1	1	1	5
**Agnelli 2012 SAVE-ONCO**	1	1	1	1	1	5
**Marayevas 2012 FRAGEM**	1	1	0 (open)	0 (open)	-	2
**Pelzer 2015 CONKO [**	1	1	0 (open)	0 (open)	-	2
**Khorana 2019 CASSINI**	1	1	1	1	1	5

Points were awarded as follows: study described as randomized, 1 point; appropriate randomization, 1 point; subjects blinded to intervention, 1 point; evaluator blinded to intervention, 1 point; description of withdrawals and dropouts, 1 point.

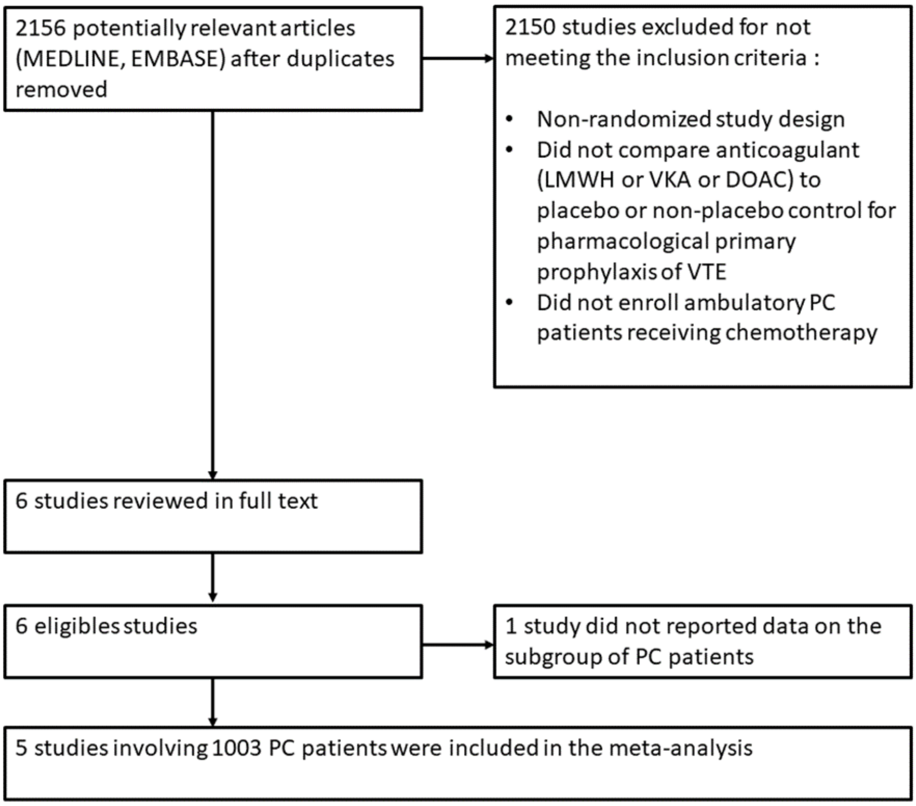

Figure S1. Search Flow Diagram.

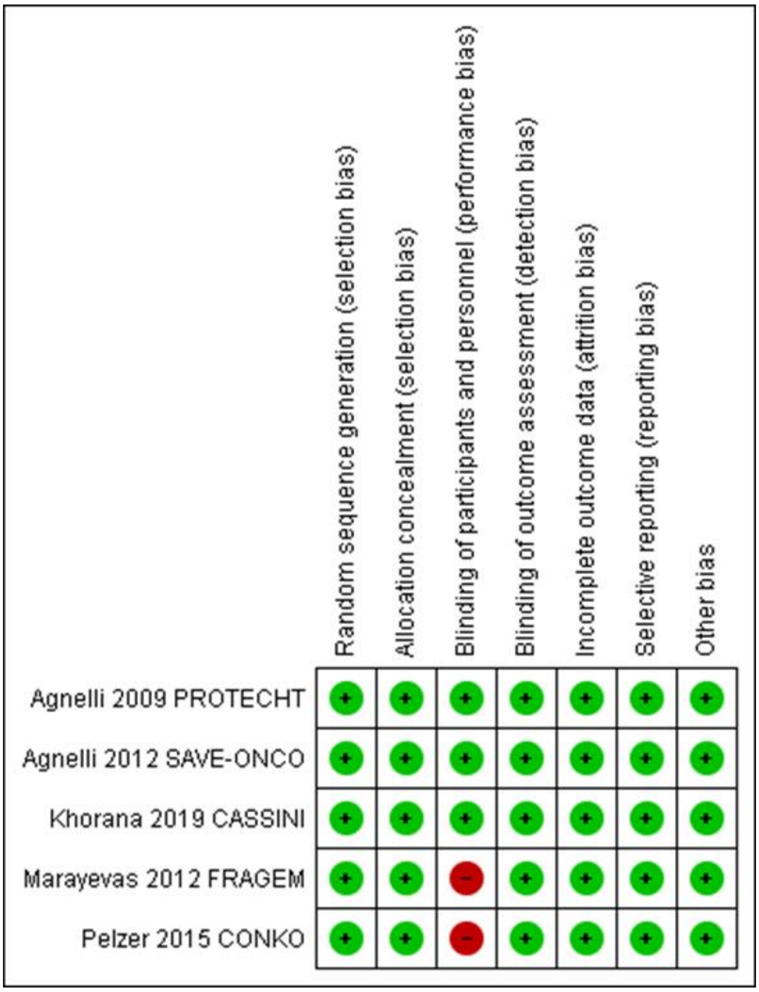

Figure S2. Quality assessment of included studies according to the Cochrane risk assessment tool.

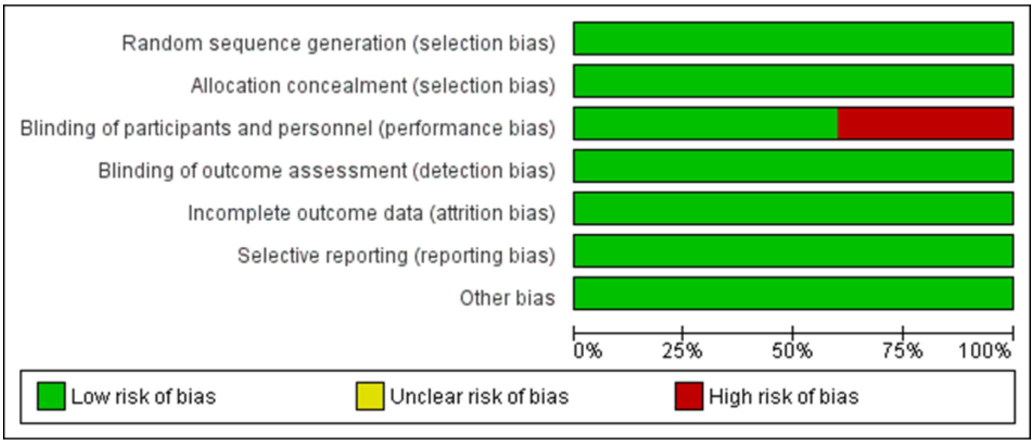

Figure S3. Risk of bias presented as percentages across all included studies according to the Cochrane risk assessment tool.

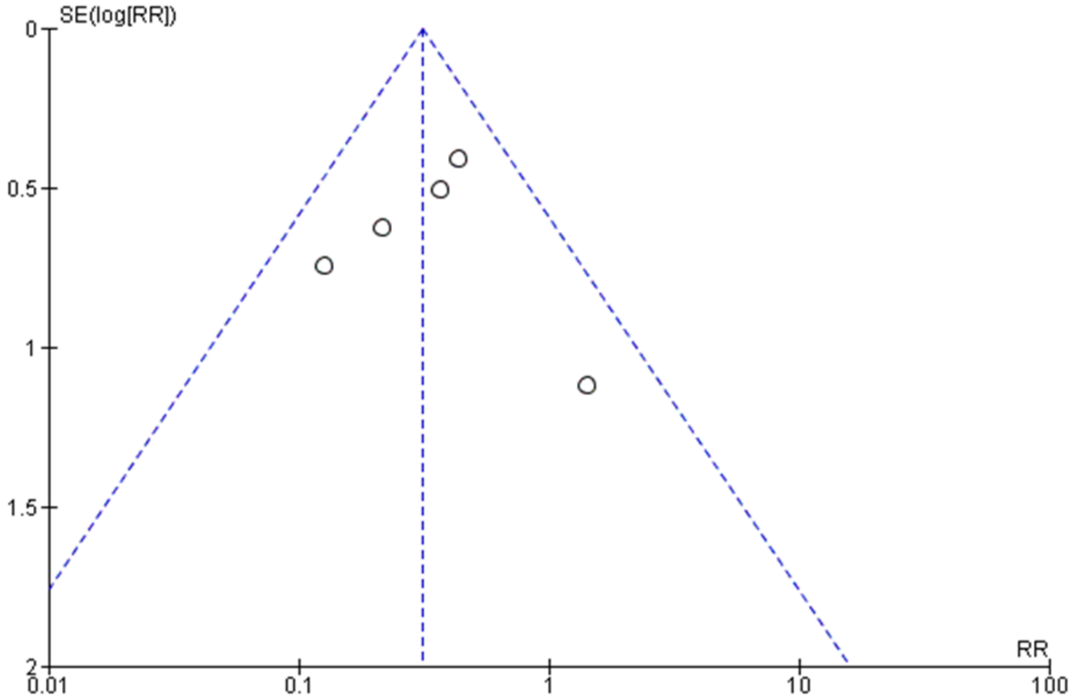

Figure S4. Funnel Plot for Venous Thromboembolism.

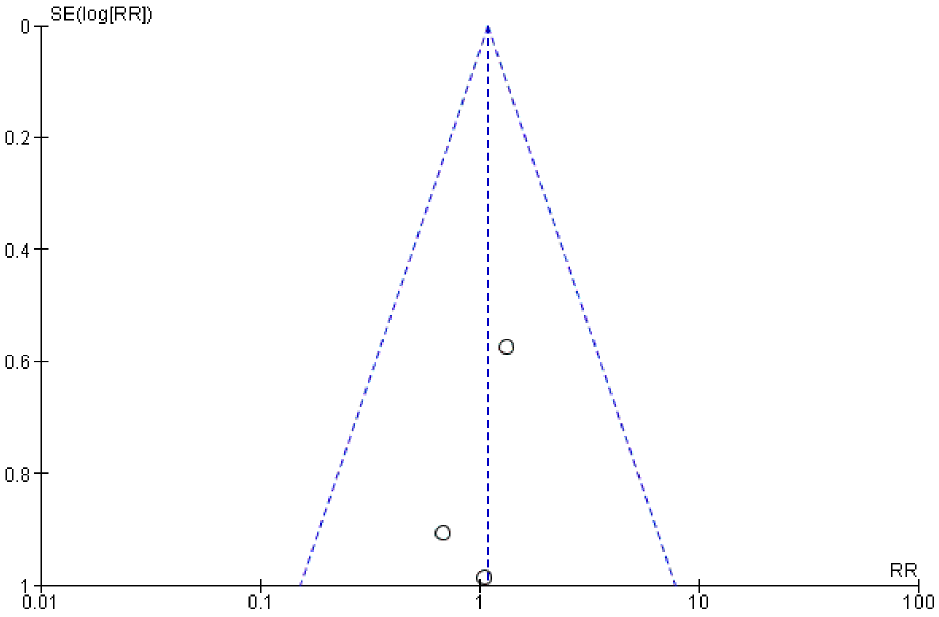

Figure S5. Funnel Plot for Major bleeding.

© 2020 by the authors. Submitted for possible open access publication under the terms and conditions of the Creative Commons Attribution (CC BY) license (http://creativecommons.org/licenses/by/4.0/).
